# Outcomes of an Acute Palliative Care Unit at a Comprehensive Cancer Center in Korea

**DOI:** 10.1089/pmr.2022.0033

**Published:** 2023-01-17

**Authors:** Si Won Lee, Jung Hye Kwon, Seung-hoon Beom, Sang Joon Shin, Hyo Song Kim, Sun Young Rha, Minkyu Jung, Joo Hyuk Sohn, Joong-Bae Ahn, Hyun Cheol Chung, Gun Min Kim, Hye Ryun Kim, Beodeul Kang, Youn Jung Hu, Hye Jin Choi

**Affiliations:** ^1^ Palliative Care Center, Yonsei Cancer Center, Seoul, Republic of Korea.; ^2^ Division of Medical Oncology, Yonsei Cancer Center, Yonsei University College of Medicine, Seoul, Republic of Korea.; ^3^ Yonsei Graduate School, Yonsei University College of Medicine, Seoul, Republic of Korea.; ^4^ Division of Hematology and Oncology, Department of Internal Medicine, Sejong Chungnam National University Hospital, Sejong, Republic of Korea.; ^5^ Division of Hematology and Oncology, Department of Internal Medicine, Chungnam National University College of Medicine, Daejeon, Republic of Korea.; ^6^ Daejeon Regional Cancer Center, Daejeon, Republic of Korea.; ^7^ Division of Medical Oncology, Department of International Medicine, CHA Bundang Medical Center, CHA University, Seongnam, Republic of Korea.

**Keywords:** acute palliative care unit, cancer, palliative care team, palliative medicine

## Abstract

**Background:**

The acute palliative care unit (APCU) bridges between active cancer treatment and hospice care. However, no study has proven the efficacy of APCU in Korea.

**Objective:**

To evaluate the first-year outcomes of the patients admitted to an APCU at a tertiary hospital in Korea.

**Design:**

The APCU admitted 205 patients between April 14, 2014, and April 30, 2015. Of these patients, 57 were evaluable for baseline and one-week follow-up Edmonton Symptom Assessment System (ESAS).

**Results:**

Of the 57 participants, 56.1% were male, with a median age of 60 years (range, 52.8–69.5 years). All patients had advanced cancer, and 42 out of 57 had terminal illnesses. The median APCU stay was 14 days (range, 10–17 days). The 42 (73.7%) patients were referred to the APCU after anticancer treatment was completed. Ten (17.5%) patients died during their stay, and 20 (35.1%) were discharged home. Among those who completed the ESAS, there were significant improvements in scores in the following symptoms: fatigue, depression, loss of appetite, and shortness of breath. Physical symptoms (pain, fatigue, nausea, drowsiness, appetite, and shortness of breath) and the total ESAS scores were significantly improved (*p* = 0.002 and *p* = 0.005, respectively). Each non-medical palliative care program, such as art and music therapy, yoga, foot massage, haircut, and body care, showed no significant differences between the group who received them and those who did not.

**Conclusion:**

During the APCU stay, the overall symptoms of inpatients were reduced. A comprehensive and multidisciplinary team approach is essential for patients who need palliative care.

## Introduction

Cancer is a leading cause of death worldwide, including in South Korea. An estimated 14.1 million new cancer diagnoses and 8.2 million cancer deaths occurred in 2012 worldwide. A total of 214,701 new cases of cancer and 76,855 cancer deaths occurred in 2015 in South Korea.^1^ Although the home is considered an ideal place of death,^2–4^ inpatient death accounts for most cases.^5,6^ Likewise, in Korea, a significantly increasing trend of hospital deaths and ∼90% of patients with cancer dying at hospitals, including hospice centers, have been noted.^5,7^

However, patients' and relatives' preferences are the leading causes of hospital death because of palliative treatments or symptom control, feelings of safety, and belief in better care.^8,9^ Adequate palliative care services for patients with cancer should be established to care for patients along the trajectory of cancer.

Palliative care has been developed in many countries. According to the WHO, the goal of palliative care is “to improve the quality of life of patients and their families facing the problem associated with life-threatening illness through the prevention and relief of suffering through early identification and impeccable assessment and treatment of pain and other issues, physical, psychosocial, and spiritual.”^10^ Palliative care is considered a broader spectrum of care than hospice care.^11^

Hospice care is confined to end-of-life care, whereas palliative care should be applied at the time of the diagnosis of the life-threatening illness and be integrated into the cancer care along with the treatment. Previously, most palliative care was used toward the end of life.^12^ After prospective randomized clinical trials with palliative care proved their efficacy in terms of overall survival, the American Society of Clinical Oncology started recommending early palliative care for advanced lung cancer and advanced cancer with severe symptoms along with active treatment.^13^

Moreover, several randomized trials showed that additional palliative care along with conventional chemotherapy improves overall survival and quality of life in patients with advanced cancer.^14–16^ The integration of early palliative care for most patients with cancer is accepted as standard practice by all oncology societies. We conducted this retrospective study to evaluate the effectiveness of acute palliative care provided at the acute palliative care unit (APCU) with patient-reported outcomes using the Edmonton Symptom Assessment System (ESAS).

Palliative care provides medical care based on patients' unmet needs, which can be estimated as patient-reported outcome measurements. The ESAS has been developed to screen patients' common symptoms in palliative settings and is widely used in clinical and research fields. We aimed at retrospectively evaluating and assessing the characteristics and discharge outcomes of patients admitted to the Yonsei Cancer Center APCU (YCC-APCU). YCC is a comprehensive cancer center that established Korea's first APCU. It opened in April 2014. This study aimed at assessing whether the APCU effectively relieved or managed patients' symptoms and at finding ways to improve outcomes further. Through this study, we hope to delineate the roles of the APCU and seek effective ways to run the unit at a tertiary hospital in Korea.

## Methods

### Study population

**FIG. 1.**
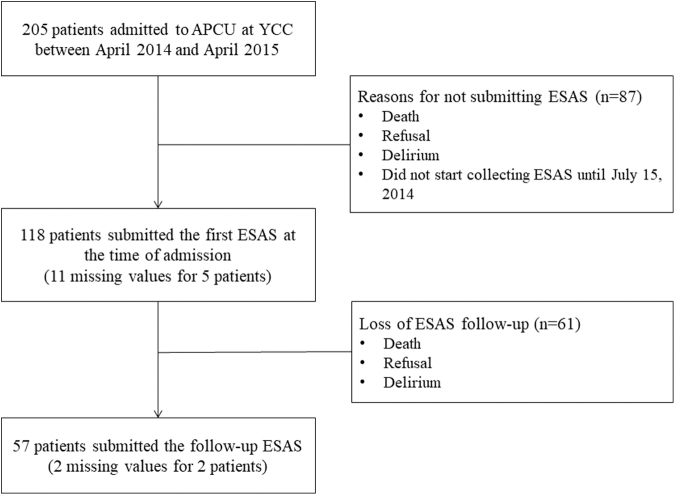
Patient selection process. APCU, acute palliative care unit; ESAS, Edmonton Symptom Assessment Scale; YCC, Yonsei Cancer Center.

This study was reviewed and approved by the institutional review board of Severance Hospital (Title: Outcomes of an APCU at a comprehensive cancer center in Korea, IRB no. 2016-2819-001). We retrospectively reviewed the medical records of consecutive patients who were admitted to YCC-APCU between April 14, 2014 and April 30, 2015 (Fig. 1). Patients were eligible if they were 20 years or older, had been diagnosed with advanced cancer, and completed the ESAS both at the time of admission and at the one-week follow-up. Patients who could not complete the symptom assessment because of impaired cognition or be discharged before their follow-up were excluded from the outcome evaluation.

### Palliative care program at YCC-APCU

The YCC-APCU consists of 12 beds and provides a palliative care program by a palliative care team that consists of a board-certified oncologist and palliative care specialist, 10 nurses who have completed a hospice education program provided by the National Cancer Center, 2 advanced practice registered nurse, an internal medicine resident, a chaplain, a social worker, a pharmacist, 2 music therapists, 2 art therapists, a yoga instructor, a volunteer coordinator, and 125 volunteers.

The team-based palliative care program included symptom management, psycho-emotional, socioeconomic, and spiritual support, art therapy such as painting and music, yoga, body care-bathing service, haircut, and foot massage by volunteers for patients in the APCU. The programs were provided to patients as deemed necessary by the palliative care team or by request of the patient or caregivers.

This program also serves as a bridge between acute care and hospice services. The ESAS has been implicated as a standard symptom screening tool in the YCC-APCU since July 2014. When a patient enters APCU, a plan is developed based on the patient's needs according to the screening tools such as ESAS, Eastern Cooperative Oncology Group (ECOG) performance status (PS), and the patient's medical condition. Subsequently, the plan is evaluated periodically.

### Assessment tools

#### Edmonton Symptom Assessment System

The ESAS has been developed to screen patients' common symptoms in palliative settings and is widely used in clinical and research fields to assess patients with cancer.^17^ It is a multidimensional tool consisting of 10 items, including pain, fatigue, nausea, depression, anxiety, drowsiness, shortness of breath, appetite, well-being, and sleep disturbances.

It was also validated for Korean patients with cancer,^18^ where each symptom measures the average intensity over the past 24 hours from 0 to 10 on a numerical scale, with 0 indicating no symptoms and 10 indicating the worst possible. According to Hui et al,^19^ the ESAS physical score (range, 0–60) is determined as a sum of pain, fatigue, nausea, drowsiness, appetite, and shortness of breath scores. The emotional score consists of symptoms of anxiety and depression (range, 0–20).

The ESAS total score was defined as the sum of the ESAS physical, emotional, and well-being scores, ranging from 0 to 90. The improvement of physical and total scores was determined by a cut-off of ≥3/60 and ≥3/90, respectively.

#### ECOG performance status

The ECOG PS was recorded at admission. The scale ranges from 0 (fully active) to 5 (dead).^20^

### Data collection

Demographics, clinical characteristics (diagnosis of cancer, anticancer treatments, and medical intervention including medications), and medical service utilization (routes of referral, source of referrals, reason for APCU admission, palliative care intervention, and discharge outcome) were collected from medical records. Prospectively collected assessment tools, including ECOG PS at baseline and ESAS score at baseline follow-up, were also obtained from medical records.

### Statistical analysis

Descriptive statistics and frequency were used for the continuous and categorical variables of patient demographics, clinical characteristics, and medical utilization. The Mann–Whitney *U*-test was used for continuous variables when comparing the ESAS-evaluated and non-evaluated groups. The chi-square test or Fisher's exact test was used for categorical variables. In addition, initial ESAS scores were compared with follow-up scores using the Wilcoxon signed-rank test. Statistical significance was set at *p* < 0.05. All statistical analyses were performed using the SPSS software (version 20.0; IBM SPSS Statistics, version 20.0 for Windows; SPSS, Inc., Chicago, IL).

## Results

### Sample characteristics

Figure 1 illustrates the patient selection process. A total of 205 patients were admitted to the YCC-APCU in the study period. Of the 205 patients, 57 completed the baseline ESAS evaluation at admission and the follow-up ESAS evaluation (Fig. 1). Demographics, clinical characteristics, and medical utilization did not differ between the study population (*n* = 57) and others (Table 1). The median age was 60 years (Q1–Q3: 52.8–69.5), and 56.1% were male.

**Table 1.** Demographics

	Total (***n*** = 205)	Group by ESAS		
		Evaluated (***n*** = 57)	Not-evaluated (***n*** = 148)	***p***
Age (years)	60 (IQR: 53.0–69.0)	60 (IQR:52.8–69.5)	59 (IQR: 53.0–68.5)	0.699
Sex (F:M)	96:109 (46.8%:53.2%)	25:32 (43.9%:56.1%)	71:77 (48.0%:52.0%)	0.709
Primary site of cancer				0.840
Hepato-biliary-pancreatic	72 (35.1%)	17 (29.8%)	55 (37.2%)	
Gastrointestinal	42 (20.5%)	12 (21.1%)	30 (20.3%)	
Lung	28 (13.7%)	9 (15.8%)	19 (12.8%)	
Gynecologic	16 (7.8%)	4 (7.0%)	12 (8.1%)	
Genito-urinary	13 (6.3%)	5 (8.8%)	8 (5.4%)	
Head and neck	5 (2.4%)	1 (1.8%)	4 (2.7%)	
Brain	5 (2.4%)	1 (1.8%)	4 (2.7%)	
Skin	4 (2.0%)	2 (3.5%)	2 (1.4%)	
Breast	3 (1.5%)	1 (1.8%)	2 (1.4%)	
Orthopedic	1 (0.5%)	1 (1.8%)	0 (0%)	
Others	16 (7.8%)	4 (7.0%)	12 (8.1%)	
ECOG Performance Status				0.926
0	2 (1%)	1 (1.8%)	1 (0.7%)	
1	57 (27.8%)	13 (22.8%)	44 (29.7%)	
2	65 (31.7%)	22 (38.6%)	43 (29.1%)	
3	43 (21.0%)	12 (21.1%)	31 (20.9%)	
4	37 (18.0%)	9 (15.8%)	28 (18.9%)	
Missing	1 (0.5%)	0	1 (0.7%)	
Route of APCU admission				0.146
Outpatient clinic	84 (41.0%)	28 (49.1%)	56 (37.8%)	
Emergency room	23 (11.2%)	3 (5.3%)	20 (13.5%)	
Inpatient clinic	98 (47.8%)	26 (45.6%)	72 (48.6%)	
Source of referral for APCU admission				0.575
Oncology	166 (81.0%)	46 (80.7%)	120 (81.1%)	
Internal medicine	13 (6.3%)	2 (3.5%)	11 (7.4%)	
Urology	9 (4.4%)	3 (5.3%)	6 (4.1%)	
Neurosurgery/Neurology	6 (2.9%)	3 (5.3%)	3 (2.0%)	
General surgery	4 (2.0%)	1 (1.8%)	3 (2.0%)	
Obstetrics/Gynecology	3 (1.5%)	0 (0%)	3 (2.0%)	
Others	4 (2.0%)	2 (3.5%)	2 (1.4%)	
Reason for APCU admission^a^				
Symptom control	193 (94.1%)	55 (96.5%)	138 (93.2%)	0.517
Terminal care	59 (28.8%)	9 (15.8%)	50 (33.8%)	0.011
Transitional care	46 (22.4%)	12 (21.1%)	34 (23.0%)	0.768
Evaluation	18 (8.8%)	2 (3.5%)	16 (10.8%)	0.098
For anticancer treatment	2 (1.0%)	1 (1.8%)	1 (0.7%)	0.480
Anticancer treatment				0.596
Ongoing	47(22.9%)	15 (26.3%)	32 (21.6%)	
Completed	158(77.1%)	42 (73.7%)	116 (78.4%)	

^a^ Numbers were duplicated per patients.

APCU, Acute palliative care unit; ECOG, Eastern Cooperative Oncology Group; ESAS, Edmonton Symptom Assessment Scale.

The most common primary site was hepatobiliary-pancreatic cancer (*n* = 17), followed by gastrointestinal (GI) (*n* = 12) and lung cancer (*n* = 9). The ECOG PS 2 or more were 43 (75.4%). The most common routes and sources of admissions were palliative consultation from the outpatient clinic (*n* = 28/57) and the oncology department (*n* = 46/57), respectively. The most common reason for APCU admission was symptom control (*n* = 54/57, 94.7%).

### Interventions during the APCU stay

Patients received individualized, patient-centered care, including anticancer treatments, symptomatic pharmacological and non-pharmacological interventions, and non-medical palliative programs at YCC-APCU (Table 2). Among the patients who had a baseline and follow-up assessment of ESAS (*n* = 57), 11 received active anticancer treatment, including palliative chemotherapy (*n* = 6) or palliative radiation (*n* = 5).

**Table 2.** Palliative Care Interventions

Interventions received at APCU^a^	No. of patients (***n*** = 57)	Total no. of patients for each category of intervention (***n*** = 205)
Anticancer treatments		
Chemotherapy	6	14
Chemoradiation	0	0
Palliative radiation	5	15
Symptomatic pharmacological interventions		
Any type of intervention on opioids	50	182
Anti-ulcer agents	45	142
Laxatives	43	136
Antipsychotics	34	103
Steroids	23	64
Anticonvulsants	21	62
Antiemetics	20	70
Aperitives	19	76
NSAIDs	14	49
Delirium medication	6	21
Antidepressants	3	21
Other analgesics	25	71
Symptomatic non-pharmacological medical intervention		
GI interventions (ERCP, EGD, colonoscopy, etc.)	3	10
Catheter interventions (Chemoport, ascites catheter, pleural catheter, etc.)	33	111
Oxygen application	25	77
Others (nebulizer, Levin tube, Foley catheter, or rectal tube)	16	59
Non-medical palliative intervention		
Art therapy	38	85
Music therapy	16	46
Yoga	8	20
Foot massage	23	62
Haircut	19	39

^a^ Each patient received multiple interventions.

EGD, Esophagogastroduodenoscopy; ERCP, endoscopic retrograde cholangio-pancreatography; EUS, Endoscopic ultrasonography; GI, gastrointestinal; NSAIDs, non-steroidal anti-inflammatory drugs.

**FIG. 2.**
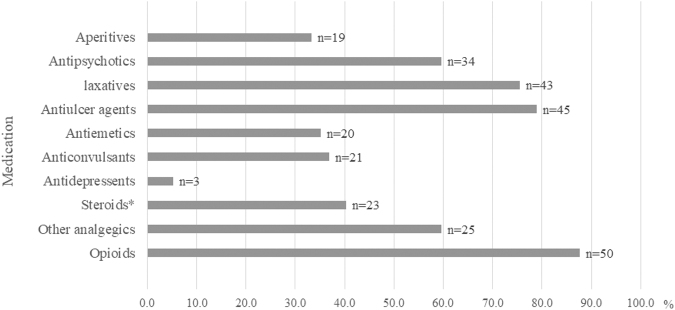
Pharmacological interventions (either initiation, discontinuation, or change of dose) during the APCU stay (*n* = 57). *The use of steroids is unclear; however, they are included as a reference.

Catheter intervention (*n* = 33) was the most common non-pharmacological medical intervention, followed by oxygen application (*n* = 25). During the APCU stay, the most commonly used medication for intervention (initiation, discontinuation, or change of dose) was opioids (*n* = 50/57, 87.7%), followed by anti-ulcer agents (*n* = 45/57, 78.9%) and laxatives (*n* = 43/57, 75.4%; Fig. 2).

### Symptom changes

The overall ESAS scores on admission and follow-up (Table 3) are summarized in terms of their mean symptom intensities. Fatigue, depression, appetite, and shortness of breath were symptoms that were reduced significantly. Physical symptoms, excluding psychological symptoms such as anxiety, were managed considerably. The total ESAS score also showed compelling evidence (*p* = 0.005) of improvement of symptoms at follow-up.

**Table 3.** ESAS Score Changes between the Time of Admission and One-Week Follow-Up

	At admission	At follow-up	***p*** ^a^
[1] Pain	5 (IQR: 3.0–7.0)	4 (IQR: 1.0–7.0)	0.076
[2] Fatigue	6 (IQR: 4.0–8.0)	5 (IQR: 2.5–7.0)	0.009
[3] Nausea	2 (IQR: 0.0–5.0)	1 (IQR: 0.0–4.0)	0.350
[4] Depression	5 (IQR: 2.0–7.5)	4 (IQR: 0.0–6.0)	0.040
[5] Anxiety	5 (IQR: 2.0–7.0)	3 (IQR: 1.0–6.0)	0.150
[6] Drowsiness	5 (IQR: 3.0–8.0)	5 (IQR: 1.0–7.0)	0.254
[7] Appetite	7 (IQR: 4.0–9.0)	5 (IQR: 2.0–8.0)	0.007
[8] Well-being	6 (IQR: 5.0–8.0)	6 (IQR: 4.0–8.0)	0.314
[9] Shortness of breath	4 (IQR: 1.0–7.0)	3 (IQR: 0.0–5.0)	0.021
[10] Sleep disturbance	5 (IQR: 1.0–7.0)	5 (IQR: 2.0–7.5)	0.373
Psychological symptom score ([4]+[5])	10 (IQR: 4.5–13.5)	7 (IQR: 2.5–12.0)	0.054
Physical symptom score ([1]+[2]+[3]+[6]+[7]+[9])	31 (IQR: 20.5–37.5)	23 (IQR: 13.0–35.0)	0.002
Total ESAS Score ([1]+ …+[9])	47.5 (IQR: 30.50–57.75)	37 (IQR: 21.5–52.5)	0.005

^a^ Wilcoxon signed-rank test.

IQR, interquartile range.

Differences in total ESAS scores by medical interventions were insignificant, except for GI interventions (Supplementary Table S1). However, only three individuals had GI interventions that could not be generalized. None of the non-medical palliative care programs—art and music therapy, yoga, foot massage, haircut, and body care—showed a significant difference between the groups who received them and did not (Supplementary Table S2).

### Discharge outcomes

Among 205 patients, discharge outcomes were compared by dividing them into 57 patients who completed the ESAS evaluation at admission and follow-up. The discharge destinations are listed in Table 4. The median length of stay for 57 patients was 14 days (range, 10–17 days), significantly longer than that of the total number of patients (10 days; range, 6.0–15.0). Eighty-one patients (39.5%) out of 205 were discharged. Of the 205 patients, 46 (22.4%) died in the APCU. The ESAS follow-up group stayed significantly longer (14 days; IQR, 10–17 days) than the group that did not complete the follow-up evaluation (9 days; IQR, 5–13.75).

**Table 4.** Discharge Outcomes

	Total (***n*** = 205)	Group by ESAS		***p***
		Evaluated (***n*** = 57)	Not evaluated (***n*** = 148)	
Destination at discharge				0.146
Death	46 (22.4%)	10 (17.5%)	36 (24.3%)	
Home	81 (39.5%)	20 (35.1%)	61 (41.2%)	
Hospice	40 (19.5%)	10 (17.5%)	30 (20.3%)	
Hospital without hospice care	29 (14.1%)	13 (22.8%)	16 (10.8%)	
Transfer	9 (4.4%)	4 (7.0%)	5 (3.4%)	
Length of stay, median (IQR)	10 (6–15)	14 (10–17)	9 (5–13.75)	<0.001

## Discussion

Typical palliative care wards focus primarily on end-of-life care similar to hospice care, whereas the APCUs concentrate more on rapid symptom control, active psychosocial treatment, shorter hospital stays, and lower inpatient mortality.^21^ As shown in Table 1, symptom control was the most common reason for admission to the YCC-APCU. Several studies have revealed improvements in symptom control when admitted to the APCU.^22–24^ Quality of life was improved or maintained.^25^ We conducted our analysis based on the assumption that symptoms and distress will be reduced after receiving palliative care in the APCU.

We analyzed the K-ESAS at baseline and at a one-week follow-up to determine if the symptoms had reduced (Table 3). The total ESAS and physical symptom scores were considerably lessened. The symptoms of fatigue, depression, appetite, and shortness of breath were significantly reduced, as shown in Table 3.

Most patients transferred to the APCU were not receiving anticancer treatment (Table 1). Most of them were referred to the YCC-APCU very late in their disease trajectory, and the majority of their unmet needs involved end-of-life care. The physician's lack of awareness about the existence of APCU might cause late referrals. Therefore, medical professionals' understanding of the need for palliative care and their ability to inform patients accurately are critical.

At the same time, late referral to APCU might be due to physicians' reluctance to send patients to the center during anticancer treatment for advanced disease. Physicians' misperception of palliative care could be one of the leading barriers to palliative care utilization,^26^ as patients and their families often assume that palliative care is provided only when no more anticancer treatments are available and is the medical care necessary at the time of death.^26^

Patients with advanced terminal cancer need appropriate, continuous multidisciplinary palliative care, as physical symptoms and psychosocial distress tend to worsen until death.^27^ Thus, the concepts of hospice and palliative care should not be confused. Hence, physicians and patients should be well aware of the benefits of palliative care.

To enhance APCU utilization, a well-organized system can be a valuable addition to palliative care promotion and physician education. Technical innovations such as the automatic referral system could be an example of development. Automatic triggering of referrals based on the patient's diagnosis, prognosis, or any need for palliative care, with timely routine symptom screening under selective predefined criteria, can be adopted.^28^

According to the change in the health status of patients, automatic consultation sent to the palliative care team allows early palliative care management. Palliative care, along with active anticancer treatment, should be available when needed.

Hui et al reported on the discharge outcome of the MD Anderson Cancer Center APCU: The median APCU stay was seven days, 36% of the patients died during their stay, 15% were discharged home, and 46% were discharged to a hospice.^29^ As presented in Table 4, the median length of APCU stay at the YCC was 10 days, the APCU death rate was 22.4%, and 39.5% were discharged home. The discharge outcomes of YCC-APCU were similar to that of the MD Anderson Cancer Center APCU.

Among the survivors who could not be discharged home (*n* = 78) from the YCC-APCU, 40 (19.5%) were transferred to various hospices (Table 4). The significantly longer stay for 57 patients who completed the ESAS evaluation at admission and follow-up, compared with the group that did not meet the follow-up evaluation (14 days vs. 9 days, *p* < 0.001), is not explanatory. We cautiously assume that patients either died or were discharged home soon after APCU admission and that follow-up was unavailable.

In our study, total care, rather than individual programs, reduced patients' symptoms. The small study population might have limitations in proving the efficacy of each program; however, overall improvement in symptoms at the APCU might reflect the need for a multidisciplinary team approach in patient care. This result is similar to other studies^12,30^ that a team approach and holistic care, rather than a specific intervention, play a role in palliative care.

### Limitations

This study had several limitations. First, the nature of a retrospective study often includes incomplete data. Second, only a tiny proportion of the study population (57 out of 205) completed the ESAS for their symptoms. Although the characteristics of the study population were not statistically different from the total patient population (*n* = 205), our results might not reflect the total population of the APCU. Finally, most of the patients (42 out of 57) failed to receive chemotherapy and were transferred to the APCU, which indicates hospice care.

This might be a reason for the limitation of the current study to present the practical role of the APCU that differs from hospice services. Our main goal is to collaborate among health care professionals with an awareness of acute palliative care.

## Conclusion

In conclusion, total care in APCU showed that patients at the end of their disease trajectory had reduced symptoms during their stay. A large-scale prospective study is needed to prove the role of APCU in improving the quality of life in patients with advanced cancer. In addition, there is a need to explore the barriers in the transition of care among health care professionals and patients.

## Supplementary Material

Supplemental data

Supplemental data

## Abbreviations Used

APCU: acute palliative care unit
ECOG: Eastern Cooperative Oncology Group
ESAS: Edmonton Symptom Assessment System
GI: gastrointestinal
PS: performance status
YCC-APCU: Yonsei Cancer Center Acute Palliative Care Unit
