# Supplementary material for: Outcomes of an Acute Palliative Care Unit at a Comprehensive Cancer Center in Korea
Source: Palliat Med Rep. 2023 Jan 17;4(1):9–16. doi: 10.1089/pmr.2022.0033 (PMC9892919; doi:10.1089/pmr.2022.0033)
Supplement: Supplemental data [file Suppl_TableS1.docx]

SUPPLEMENTAL TABLE 1. DIFFERENCES IN TOTAL ESAS SCORES BY

NON-PHARMACOLOGICAL MEDICAL INTERVENTION

| *Medical interventions* | *Received* | *Not received* | P *value* |
| --- | --- | --- | --- |
| Radiation therapy | -6.0 (IQR: -11.5~9.3)  -2.0 (IQR: -11.5~9.0) | -7.0 (IQR: -18.0~3.0)  -5.0 (IQR: -13.0~1.0) | .582  .507 |
| Chemotherapy | -8.0 (IQR: -14.0~-2.0)  -8.5 (IQR: -13.0~-2.0) | -6.0 (IQR: -16.8~3.8)  -6 (IQR: -13.0~1.8) | .765  .658 |
| GI interventions  (ERCP, EGD, EUS, colonoscopy, etc.) | 11.0  2.0 | -8.5 (IQR: -17.0~3.0)  -6.0 (IQR: -13.0~1.0) | .047  .120 |
| Catheter interventions  (chemo-port, ascites catheter, pleural catheter, etc.) | -6.0 (IQR: -19.8~9.3)  -3.0 (IQR: -13.3~7.0) | -7.0 (IQR: -14.5~-0.5)  -7.0 (IQR: -13.0~-1.0) | .616  .378 |
| Oxygen application | -2.0 (IQR: -12.5~5.8)  -3.0 (IQR: -9.3~4.8) | -11.0 (IQR: -20.5~-4.0)  -9.5 (IQR: -14.0~-0.5) | .166  .166 |
| Others (nebulizer, foley, Levin, or rectal tubes, etc.) | -6.0 (IQR: -21.0~9.5)  -1.0 (IQR: -12.5~7.0) | -8.0 (IQR: -15.3~1.0)  -5.0 (IQR: -13.3~0.3) | .742  .303 |

Upper values are differences in total ESAS scores, and lower values are differences in physical ESAS.

ESAS, Edmonton Symptom Assessment Scale; GI, Gastrointestinal;

ERCP, Endoscopic retrograde cholangio-pancreatography; EGD, Esophagogastroduodenoscopy;

EUS, Endoscopic ultrasonography
