# Supplementary material for: Outcomes of an Acute Palliative Care Unit at a Comprehensive Cancer Center in Korea
Source: Palliat Med Rep. 2023 Jan 17;4(1):9–16. doi: 10.1089/pmr.2022.0033 (PMC9892919; doi:10.1089/pmr.2022.0033)
Supplement: Supplemental data [file Suppl_TableS2.docx]

SUPPLEMENTAL TABLE 2. DIFFERENCES IN TOTAL ESAS SCORES BY

NON-MEDICAL PALLIATIVE CARE PROGRAM

| *Non-medical palliative care program* | *Received* | *Not received* | P *value* |
| --- | --- | --- | --- |
| Art therapy | -7.0 (IQR: -16.0~3.0)  -4.5 (IQR: -13.0~1.0) | -6.0 (IQR: -20.0~3.8)  -7.0 (IQR: -13.0~3.3) | .960  .786 |
| Music therapy | -13.0 (IQR: -22.5~1.5)  -10.0 (IQR: -15.0~0.0) | -4.0 (IQR: -14.0~4.3)  -4.0 (IQR: -12.3~2.5) | .158  .244 |
| Yoga | -11.5 (IQR: -21.0~4.5)  -8.5 (IQR: -14.0~4.0) | -6.0 (IQR: -15.5~3.3)  -5.0 (IQR: -13.0~1.3) | .581  .909 |
| Foot massage | -12.0 (IQR: -16.8~0.0)  -7.0 (IQR: -14.3~-0.3) | -3.0 (IQR: -15.0~8.0)  -4.0 (IQR: -13.0~7.0) | .341  .547 |
| Haircut | -9.0 (IQR: -18.3~4.5)  -5.0 (IQR: -13.8~3.3) | -5.0 (IQR: -15.0~3.0)  -5.0 (IQR: -13.0~1.0) | .832  .678 |
| Body care | -12.0 (IQR: -20.5~-1.0)  -8.0 (IQR: -14.5~-1.0) | -2.0 (IQR: -12.5~8.3)  -3.0 (IQR: -13.0~7.0) | .053  .244 |

Upper values are differences in total ESAS scores, and lower values are differences in physical ESAS scores.

ESAS, Edmonton Symptom Assessment Scale
